# Characteristics of participants in a public rubella antibody testing program conducted at a Japan professional football league venue

**DOI:** 10.1002/jgf2.611

**Published:** 2023-02-16

**Authors:** Toshinori Nishizawa, Kuniyoshi Hayashi, Noriyuki Amano, Gautam A. Deshpande, Hiroko Arioka

**Affiliations:** ^1^ Division of General Internal Medicine St. Luke's International Hospital Tokyo Japan; ^2^ Department of Global Health Promotion Tokyo Medical and Dental University Tokyo Japan; ^3^ Institute of Religion and Culture Kyoto Women's University Kyoto Japan; ^4^ College of Nursing St. Luke's International University Tokyo Japan; ^5^ Amano Clinic Saitama Japan; ^6^ Department of General Medicine Juntendo University Tokyo Japan

**Keywords:** behavioral factors, infectious diseases, Japan's professional football league, public health campaign, rubella antibody tests

## Abstract

**Background:**

Hypothesizing that soccer‐associated public health campaigns influence men more than women, we investigated the characteristics and motivations of participants who received rubella antibody testing at a Japanese professional football league event.

**Methods:**

This was a survey‐based cross sectional study, comparing the characteristics and motivations between men and women regarding rubella antibody testing.

**Results:**

Free and convenient testing was the biggest behavioral influencer, but the information provided by healthcare professionals and athletes also played a strong motivating role. Men reported more influence from celebrity athletes than women.

**Conclusions:**

Public health attention raised by celebrity athletes may facilitate rubella awareness among male spectators.

## 1 INTRODUCTION

Japan experienced an outbreak of rubella between 2012 and 2013, resulting in 17,000 cases of rubella and 45 cases of congenital rubella syndrome.^1^ 5252 cases of rubella and five cases of congenital rubella syndrome were reported in the latest 2019 latest outbreak,^2^ most cases were men who had not received routine rubella vaccination during childhood. In April 2019, the Japanese Ministry of Health, Labor and Welfare (MHLW) launched a program providing free rubella antibody testing to susceptible men born between 1962 and 1978, including free vaccinations for those with low antibody levels. This countermeasure was initially a 3‐year plan with the goal of increasing the population prevalence of rubella antibodies to >90% by March 2022.^3^ However, by 2022, only 26.9% of those eligible had been tested for antibodies, with 5.9% of those eligible receiving vaccinations through this program,^4^ prompting the MHLW to extend the program through March 2025.^5^

Public health awareness raised by sports events and celebrities might be effective but remains an untested strategy in Japan.^6^, ^7^ Spectators of Japan's professional football league (J‐League) are mostly men in their 40s (26.9%) and 50s (20.5%) with an average age of 42.8 years old, a group of high‐risk for low rubella antibody titers.^8^ In addition, 10%–30% of women of childbearing age do not have antibody titers considered sufficient for pregnancy.^9^ In collaboration with J‐League, we provided rubella awareness activities before an official match, followed by free and voluntary rubella antibody testing for adult spectators. As part of this effort, Japanese celebrity soccer players assisted with rubella awareness activities via social networking platforms and print media.

As free public health testing at sports events is exceedingly rare in Japan, we surveyed the characteristics of spectators who received rubella antibody tests, exploring the behavioral influencers behind their motivation to receive testing. Hypothesizing that sports‐associated campaign influenced men more than women, we also investigated whether there were gender differences in knowledge and motivations related to rubella.

## 2 METHODS

### 2.1 Respondents

Eighty‐nine adult spectators underwent rubella antibody tests at the J‐League FUJI XEROX SUPER CUP 2020 held on February 8, 2020,^10^ to whom we mailed questionnaires and study consent forms, asking them to return the forms upon completion. The study protocol was approved by the Ethics Committee of St. Luke's International Hospital, Japan (Number: 19‐R213).

### 2.2 Questionnaires

We asked participants about their educational background, frequency of health care visits, and presence of partners and children. Using a four‐point scale, we assessed participants' knowledge of rubella, congenital rubella syndrome, and rubella vaccine. Behavioral influencers that potentially influenced participants to receive a rubella antibody test were divided into five categories, each assessed on a four‐point scale: (1) testing was free and convenient; (2) testing was recommended by a family member; (3) testing was recommended by a friend; (4) healthcare providers explained to me about rubella in front of the venue; and (5) celebrity soccer players disseminated information about rubella through SNS and posters. Details of the questions are provided in Table 1.

**TABLE 1:** Characteristics of Japan's professional football league spectators (*n* = 38) receiving rubella antibody testing, and differences between men and women.

Variables	ALL (*n* = 38)	Men (*n* = 26)	Women (*n* = 12)	*p* Value
Age (mean, range)	40.0 (28–56)	41.4 (28–56)	38.3 (30–56)	0.30
	*N* (%)			
Gender				
Male	26 (68.4)	26 (100)	0 (0)	
Rubella antibody level (The hemagglutination assay method)				
≤8:1	6 (15.8)	5 (19.2)	1 (8.3)	0.64
>8:1	32 (84.2)	21 (80.8)	11 (91.7)	
**Social characteristics**				
Highest education achieved				
Graduate school	3 (7.9)	3 (11.5)	0 (0)	0.14
University	26 (68.4)	19 (73.1)	7 (58.3)	
Junior college	2 (5.2)	0 (0)	2 (16.7)	
Vocational technical school	5 (13.2)	3 (11.5)	2 (16.7)	
High school	2 (5.3)	1 (3.8)	1 (8.3)	
Junior high school	0 (0)	0 (0)	0	
Frequency of healthcare visits				
Regular visits to primary care and annual health examination	10 (26.3)	7 (26.9)	3 (25)	1
Irregularly visit primary care and annual health examination	4 (10.5)	3 (11.5)	1 (8.3)	
Have no primary care but receive annual health examination	2 (5.3)	1 (3.8)	1 (8.3)	
Have no primary doctor and do not receive annual health examination	22 (57.9)	15 (57.7)	7 (58.3)	
Partner				
Yes	24 (63.2)	18 (69.2)	6 (50)	0.30
Children				
Yes	13 (34.2)	10 (38.5)	3 (25)	0.49
**Rubella Health Literacy**				
Rubella				
I know that it is prevalent and am familiar with its symptoms	11 (29.0)	5 (19.2)	6 (50)	0.10
I know that it is prevalent	14 (36.8)	12 (46.2)	2 (16.7)	
I have heard its name	13 (34.2)	9 (34.6)	4 (33.3)	
I do not know anything about it	0 (0)	0 (0)	0 (0)	
Congenital rubella syndrome				
I know that pregnant women infected with rubella can transmit the disease to their babies, and also know the symptoms that these babies have	3 (7.9)	0 (0)	3 (25)	<0.05
I know that pregnant women infected with rubella can transmit the disease to their babies	19 (50)	12 (46.2)	7 (58.3)	
I have heard of its name	0 (0)	0 (0)	0 (0)	
I do not know anything about it	16 (42.1)	14 (53.8)	2 (16.7)	
Rubella vaccine				
I have received a rubella antibody test or vaccination as an adult	2 (5.3)	1 (3.8)	1 (8.3)	0.29
I know that even adults can receive rubella antibody tests or vaccination	15 (39.5)	8 (30.8)	7 (58.3)	
I know there is a rubella vaccine	17 (44.7)	14 (53.8)	3 (25.0)	
I do not know anything about it	4 (10.5)	3 (11.5)	1 (8.3)	
**Behavioral influencers**				
Testing was free and convenient				
Strongly agree	30 (79.0)	18 (69.2)	12 (100)	0.11
Agree	5 (13.2)	5 (12.2)	0 (0)	
Disagree	3 (7.9)	3 (11.5)	0 (0)	
Strongly disagree	0 (0)	0 (0)	0 (0)	
Recommended by family member				
Strongly agree	6 (15.8)	5 (19.2)	1 (8.3)	0.84
Agree	6 (15.8)	3 (11.5)	3 (25.0)	
Disagree	4 (10.5)	3 (11.5)	1 (8.3)	
Strongly disagree	16 (42.1)	11 (42.3)	5 (41.7)	
Missing	6 (15.8)	4 (15.4)	2 (16.7)	
Recommended by a friend				
Strongly agree	3 (7.9)	3 (11.5)	0 (0)	0.82
Agree	4 (10.5)	2 (7.7)	2 (16.7)	
Disagree	3 (7.9)	2 (7.7)	1 (8.3)	
Strongly disagree	17 (44.7)	12 (46.2)	5 (41.7)	
Missing	11 (29.0)	7 (26.9)	4 (33.3)	
Healthcare providers explained about rubella (“Information provided by healthcare professionals”)				
Strongly agree	11 (29.0)	10 (38.5)	1 (8.3)	0.094
Agree	22 (57.9)	13 (50.0)	9 (75.0)	
Disagree	3 (7.9)	1 (3.8)	2 (16.7)	
Strongly disagree	2 (5.3)	2 (7.7)	0 (0)	
Celebrity soccer players disseminated information about rubella via SNS and posters (“Information provided by celebrity soccer players”)				
Strongly agree	8 (21)	8 (30.8)	0 (0)	<0.05
Agree	17 (44.7)	10 (38.5)	7 (58.3)	
Disagree	9 (23.7)	4 (15.4)	5 (41.7)	
Strongly disagree	4 (10.5)	4 (15.4)	0 (0)	

*Note*: The hemagglutination assay (HI) method was used to determine antibody titers against rubella (Denka Seiken Co, Ltd, Tokyo). Cutoff antibody titers for rubella were defined as ≦1:8 (on the HI assay). We sent the results of antibody titers to all participants and recommended those with negative results to receive rubella vaccination at a nearby medical institution. We compared the baseline characteristics between gender using the Fisher's exact tests for categorical variables and the Mann–Whitney test for continuous variables. A statistical significance was set as *p* < 0.05.

### 2.3 Statistical analysis

Descriptive statistics were used to summarize the data. We compared the baseline characteristics between genders using Fisher's exact tests for categorical variables and the Mann–Whitney test for continuous variables. Statistical significance was set as *p* < 0.05. All statistical analysis was performed using R Statistical Software (version 3.4.1; R Foundation for Statistical Computing).

## 3 RESULTS

Of 89 testing recipients, 38 (26 men; 68%) returned a completed questionnaire and consented to the study (response rate, 43%). The age of all respondents ranged from 28 to 56 years, with a mean of 40.0 years. Twenty‐nine (76%) had a college education or higher, 24 (63%) had a partner, and 13 (34%) had children. With regard to knowledge of rubella, respondents were divided among those who had heard of it (34%), knew that it was prevalent (37%), and knew about both the outbreak and its symptoms (29%). Ninety‐two percent of respondents considered “free and convenient” as an important factor for testing. Eighty‐seven percent of respondents reported influence from information provided by healthcare professionals, and 65.7% reported influence from information provided by celebrity soccer players. By gender, men reported less knowledge of congenital rubella syndrome and demonstrated more influence from celebrity soccer players than women. There were no statistically significant differences between men and women regarding knowledge of rubella and its vaccine, or other behavioral influencers (Table 1).

## 4 DISCUSSION

Although this study is not intended to show the facilitating factors for taking the antibody test in subjects of the 5th phase rubella routine immunization program, but rather in the general population, free rubella antibody testing and information provided by healthcare professionals proved to be useful for most participants. The fact that men tended to report more influence from celebrity soccer players may help raise rubella awareness for male spectators in the J‐League. Working with celebrity athletes, medical professionals might be able to have a positive impact on public health attention.^11^

A previous study examining factors of participation in the rubella catch‐up campaign in Japan found that male respondents who understood the government's recommendations were more likely to receive rubella antibody testing.^12^ Another study of Japanese women showed that knowledge of rubella was one predictor of self‐reported rubella vaccination status.^13^ We also showed that men were less aware of congenital rubella syndrome. These studies suggested that, in order to promote antibody testing and vaccination, population‐wide increases in infectious disease literacy are important. To this end, awareness‐raising and educational activities against rubella continue to be needed.

Regarding limitations of our study, the response rate was low with a consequently small sample size; the interpretation of these results may be influenced by multiple biases, including observer and responder bias, in this exploratory research.

## 5 CONCLUSION

Free and convenient testing was the biggest influence, but the information provided by healthcare professionals and celebrity athletes were also major motivation, for spectators' getting antibody tests at a sports event. Celebrity athletes' influence might be especially influential among male spectators.

## FUNDING INFORMATION

The author TN was supported by research funding by LINK‐J SCOOP 2019 (200 thousand yen) and ANA Wonder FLY (372 thousand yen).

## CONFLICT OF INTEREST STATEMENT

This event was funded by LINK‐J SCOOP 2019 and ANA Wonder FLY. Dr. Nishizawa was supported by research funding by LINK‐J SCOOP 2019 (200,000 JPY) and ANA Wonder FLY (372,000 JPY). No other authors reported any financial disclosures.

## ETHICAL APPROVAL

None.

## PATIENT CONSENT STATEMENT

Written informed consent was obtained from all individual participants included in the study.
